# Treatment outcomes of visceral leishmaniasis in Ethiopia from 2001 to 2017: a systematic review and meta-analysis

**DOI:** 10.1186/s40249-018-0491-7

**Published:** 2018-10-19

**Authors:** Eyob Alemayehu Gebreyohannes, Akshaya Srikanth Bhagvathula, Tadesse Melaku Abegaz, Mohammed Assen Seid

**Affiliations:** Department of Clinical Pharmacy, College of Medicine and Health Sciences, University of Gondar, Gondar, Ethiopia

**Keywords:** Visceral leishmaniasis, Sodium stibogluconate, Amphotericin b, Paromomycin, Treatment, Success, Mortality, HIV/AIDS, Ethiopia

## Abstract

**Background:**

Ethiopia has the highest number of visceral leishmaniasis (VL) cases after Sudan in Sub-Saharan Africa. However, there was lack of comprehensive data on VL treatment outcome despite the huge burden of the diseases in the country. Hence, we aimed to perform a systematic review and meta-analysis on this topic to obtain stronger evidence on treatment outcomes of VL from the existing literature in Ethiopia.

**Methods:**

The Cochrane guidelines to conduct meta-analysis following the Preferred Reporting Items for Systematic review and Meta-Analysis statement was used to conduct a computerized systematic search of the PubMed, Google Scholar, and ScienceDirect databases. Random effects model was used to combine studies showing heterogeneity of Cochrane *Q P* < 0.10 and *I*^*2*^ > 50. Treatment outcomes were assessed at end of treatment and at 6 months follow-up. Subgroup analyses were performed on treatment outcomes based on the different antileishmanial treatment options and patients’ HIV status.

**Results:**

Fifteen studies were included in the final analyses. At end of treatment, an overall treatment success rate of 82.6% was noticed. At 6 months follow-up, the overall treatment success rate was 72.2%. For patients treated with sodium stibogluconate (SSG), the treatment success rates at the end of treatment and at six-month follow-up were 81.5% and 80.7%, respectively. Multiple doses of liposomal-amphotericin B (L-AMB) had treatment success rates of 96.7 and 71–100% at the end of treatment and at 6 months follow-up, respectively. The combination of SSG with paromomycin (PM) gave treatment success rates of up to 90.1% at the end of treatment. HIV-infected individuals were found to have a higher mortality (*odds ratio* = 4.77, 95% *CI*: 1.30–17.43, *P* = 0.009) rate at 6 months follow-up.

**Conclusions:**

SSG alone has shown lower treatment efficacy in the management of VL when compared to combination of SSG with PM and multiple doses of L-AMB. The combination of SSG with PM gave good treatment success rates with shorter duration of treatment. Hence, the combination of SSG with PM should be used preferentially over SSG monotherapy. Multiple doses of L-AMB showed great efficacy especially among patients with complications, severe disease, HIV co-infection, and intolerance to the adverse effects of antimonials. HIV-infected individuals had a worse prognosis than their HIV-negative counterparts.

**Electronic supplementary material:**

The online version of this article (10.1186/s40249-018-0491-7) contains supplementary material, which is available to authorized users.

## Background

Visceral Leishmaniasis (VL), also known as ‘kala azar’, is a vector-borne protozoan disease [1]. Considered among neglected tropical diseases, VL is the most severe form (fatal if untreated) of leishmaniases, characterized by weight loss, fever, splenomegaly, hepatomegaly and/or lymphadenopathies, and anemia [1–4]. An estimated 500 000 people are affected with VL worldwide [2]. In sub-Saharan Africa, Ethiopia has recorded the highest number of VL cases after Sudan with more than 4000 people registered for treatment every year [5, 6].

The primary etiologic agent for VL, particularly in East Africa and India, is *Leishmania donovani* complex. On the other hand, *Leishmania infantum* affects Europe, North Africa and Latin America [1]. In low and middle-income countries, poverty is a major underlying determinant and potentiating factor of leishmaniasis. It can increase morbidity, disease progression, and mortality mainly through poor nutritional status [7].

During the last three decades, VL has become one of the most important opportunistic infection in HIV-infected patients. The highest prevalence (20–30%) of HIV-VL co-infection has been reported from Northwest Ethiopia [8]. In the absence of highly active antiretroviral therapy, VL patients with HIV will have poor prognosis and higher relapse rates (close to 100%) even after effective antileishmanial treatment [8–10].

Antileishmanial drugs such as liposomal amphotericin B (L-AMB), pentavalent antimonial drugs, including sodium stibogluconate (SSG) and meglumine antimoniate (MA), paromomycin sulfate (PM), and miltefosine showed therapeutic efficacy against VL. In Ethiopia, a combination of SSG and PM given for 17 days has been considered as the first-line treatment for VL. However, in cases of treatment failure, relapse, and severe toxicity cases L-AMB is recommended as second-line treatment [6, 8, 11]. Several studies conducted among VL patients across the world showed poor treatment outcomes [12–14] and the current arsenal used to fight VL is relatively old chemotherapies. In particular, a better understanding of treatment outcomes in Ethiopia could help national and international organizations to develop strategies for improving outcomes in VL patients. Hitherto, no systematic review and/or meta-analysis on this topic was identified from Ethiopia. Hence, we aimed to perform a systematic review and meta-analysis on this topic to obtain stronger evidence on treatment outcomes of VL from the existing literature in Ethiopia.

## Methods

### Search strategies

The Cochrane guidelines to conduct meta-analysis following the Preferred Reporting Items for Systematic review and Meta-Analysis (PRISMA) statement [15] was used to conduct a computerized systematic search of the PubMed, Google Scholar, and ScienceDirect databases. Both observational (prospective and retrospective) and interventional studies were included in the review using the following Medical Subject Headings (MeSH) terms: “(((visceral leishmaniasis OR leishmaniasis OR kala-azar)) AND (treatment OR management OR sodium stibogluconate OR meglumine antimoniate OR pentamidine OR amphotericin b OR paromomycin OR pentavalent antimonials OR antimonials OR miltefosine)) AND Ethiopia”. Only studies conducted in Ethiopia were included in the study; however, in cases where studies were conducted in Ethiopia as well as in other countries, the data obtained from Ethiopia only was extracted. Publication dates were not used as inclusion or exclusion criteria and research papers published before November 30, 2017 were included.

### Inclusion criteria

Papers fulfilling the following criteria were included in the study: studies presented as original articles; studies that examined VL treatment outcomes; studies, exclusively or as part of a larger study, conducted in Ethiopian population; and studies written in English.

### Exclusion criteria

The following papers were excluded from the study: studies whose full articles were not available online whether for free or with subscription (payment); studies that didn’t specify type of antileishmanial drugs used; case reports; in vitro and animal models; and studies that assessed prophylaxis against VL and rates of treatment relapse (Additional file 1).

### Review process

**Fig. 1:**
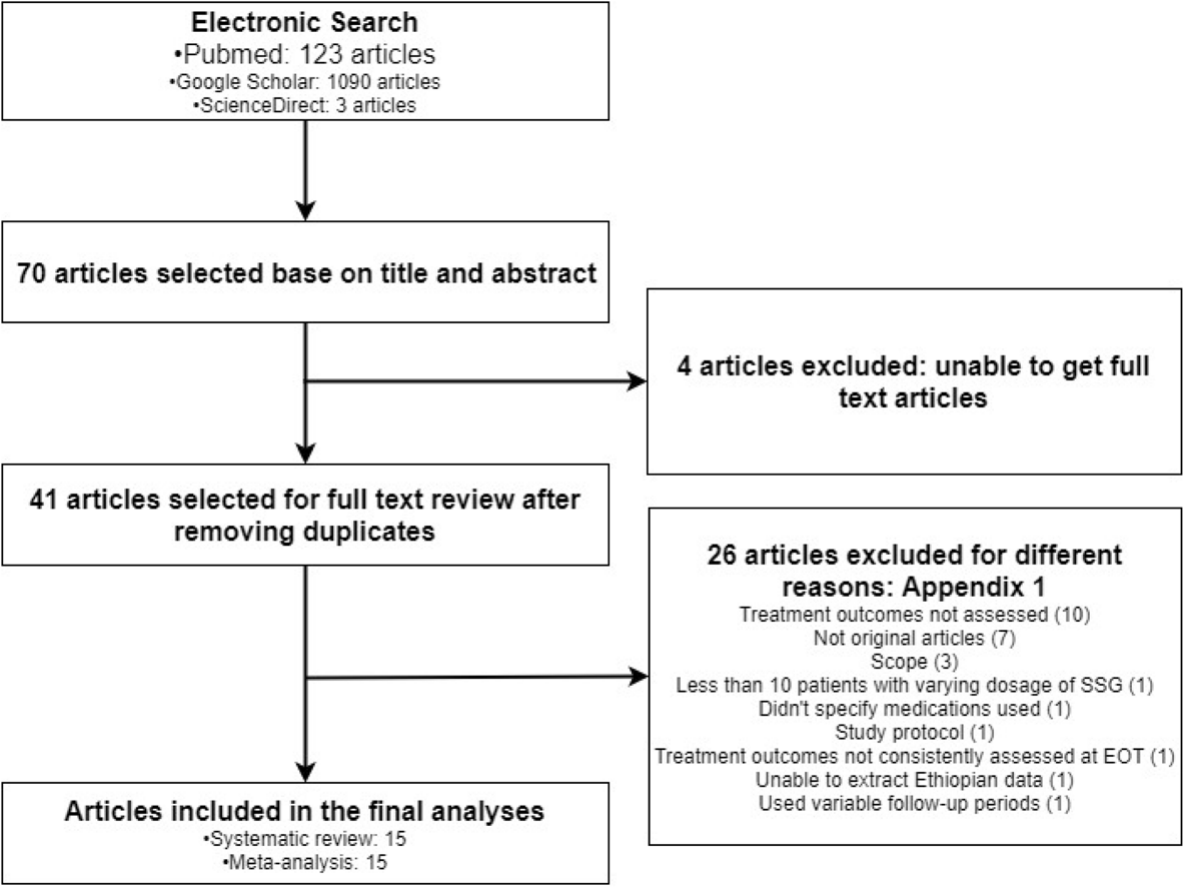
Flow diagram showing the number of articles identified in the systematic review and meta-analysis on visceral leishmaniasis treatment outcomes in Ethiopia

Two reviewers (EAG and TMA) independently screened the three electronic databases (PubMed, Google Scholar, and ScienceDirect) to identify potentially eligible studies based on their titles and abstracts. All the research articles that were identified from searches of the electronic databases were imported into the EndNote software version ×5 (Thomson Reuters, USA) and duplicates were removed. Studies that were potentially eligible were selected for fulltext review. Before data extraction had begun, full-length articles of the selected studies were read to confirm for fulfilling the inclusion criteria. Then data was extracted from full-length articles who fulfilled the inclusion criteria. Discrepancies were resolved by an independent review from the third researcher (ASB) and mutual consent after discussion (Fig. 1).

### Data extraction

Data on the types of study design, the year the studies were conducted, length of study, and geographic location of the study area was first extracted. Mean and median ages, sex, and HIV status of study participants were then extracted. Finally, data regarding the types of antileishmanial agents, treatment outcome measures (including treatment success rates, treatment failure rates, and mortality rates both at the end of treatment and at 6 months follow-up), and treatment duration were extracted to be included in the systematic review and meta-analysis. Patients who defaulted or were missing from the study were excluded from the final analyses.

### Operational definitions

*Treatment success at the end of treatment (EOT)*: cure as evidenced by clinical improvement and/or absence of parasite in tissue aspirates once patients complete antileishmanial treatment regimen.

*Treatment failure at EOT*: lack of improvement or worsening of clinical signs and symptoms and/or failing to clear the parasite of tissue aspirates once patients complete antileishmanial treatment regimen.

*Mortality at EOT*: number of patients who died just after completing or while taking the antileishmanial treat regimen.

*Treatment success at 6 months follow-up (6MFU)*: treatment success at EOT plus cure as evidenced by clinical improvement and/ or absence of parasite in tissue aspirates or absence of disease relapse 6 months after completion of antileishmanial treatment regimen.

*Treatment failure at 6MFU*: cumulative number of patients with treatment failure at EOT and lack of improvement or worsening of clinical signs and symptoms and/or failing to clear the parasite of tissue aspirates or the presence of disease relapse 6 months after completion of antileishmanial treatment regimen.

*Mortality at 6MFU*: cumulative number of patients who died at EOT and within 6 months of completing antileishmanial treatment regimen.

### Quality assessment

Two reviewers (EAG and TMA) independently assessed the methodological quality of studies using Strengthening the Reporting of Observational Studies in Epidemiology (STROBE) statement for observational studies [16] and modified Jadad scale for randomized controlled trials (RCTs) [17]. The scores for the modified Jadad scale can range from 0 to 8 (low to high quality). Scores of 4–8 represent good to excellent, whereas 0–3 represents low or poor quality. Similarly, for the observational studies scores over 75% using the STROBE checklist were considered as having high quality. The STROBE checklist has 22 items and studies that fulfill at least 17 out of the 22 criteria (> 75%) were considered to have high quality.

### Statistical analysis

OpenMetaAnalyst software (www.cebm.brown.edu/openmeta) was used to perform the meta-analysis. The Cochrane *Q* and the *I*^*2*^ were used to evaluate heterogeneity of studies. *Q* test indicates the presence of heterogeneity while *I*^*2*^ shows the degree of heterogeneity. As studies with Cochrane *Q P* < 0.10 and *I*^*2*^ > 50 were considered to have high heterogeneity, random effects model was used to combine these studies. Subgroup analyses were performed on treatment outcomes based on the different antileishmanial treatment options and patients’ HIV status across different studies.

## Results

Our search identified 1216 citations from three electronic databases (PubMed, Google Scholar, and ScienceDirect). Of these, 41 full-text articles were reviewed for eligibility. Fifteen studies [18–32] fulfilled the inclusion criteria and were included in the final analyses (Fig. 1). There were 10 observational [18–27] and 5 interventional studies [28–32] with a sample size ranging from 54 [30] to 2177 [20]. Most of the studies [18–26, 28–30] were conducted entirely in Ethiopia while three of the studies [27, 31, 32] were multicenter, where some study participants were from other countries (Additional file 2). All studies were hospital-based and except two studies [25, 32], all included HIV-infected individuals [18–24, 26–31]. In total, there were 5852 participants of which 2444 were male and 153 female patients (unable to determine sex for 3255 patients). Seven studies [18, 23, 24, 26, 28, 29, 31] included 2083 VL patients who were treated with SSG and while the rest of patients were treated with L-AMB (512 patients) [22, 24, 25, 32], combination of SSG and PM (310 patients) [24, 27], miltefosine (281 patients) [29], PM (75 patients) [31], and MA (54 patients) [30].

### VL treatment success

**Fig. 2:**
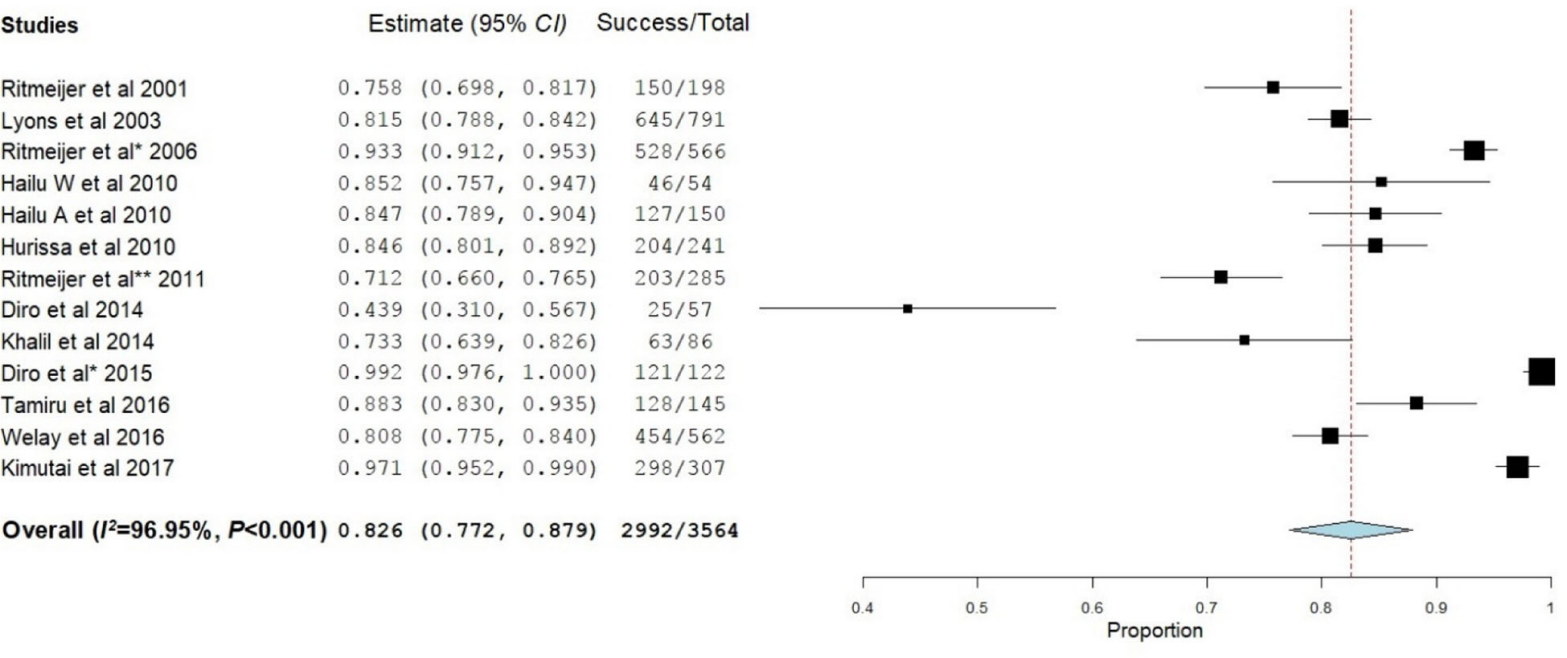
Treatment success of visceral leishmaniasis patients at the end of the treatment

**Fig. 3:**
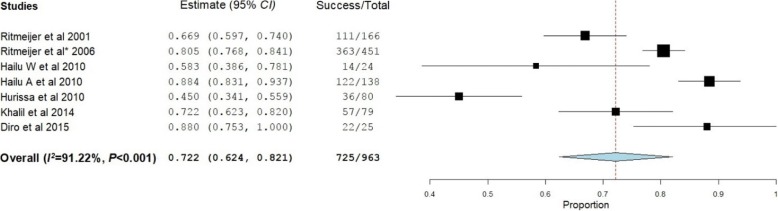
Treatment success of visceral leishmaniasis patients at six months of follow-up

Treatment successes were assessed at two different times: at EOT and at 6MFU. Fourteen [18, 20–32] and eight studies [19, 21, 24, 28–32] reported treatment outcomes at EOT and at 6MFU, respectively. At EOT, an overall treatment success rate of 82.6% [(95% *CI*: 77.2–87.9%), *P* < 0.001] was noticed (Fig. 2). At 6MFU, the overall treatment success rate decreased to 72.2% [(95% *CI*: 62.4–82.1%), *P* < 0.001] (Fig. 3).

Treatment success rate at EOT was 81.5% (95% *CI*: 72.8–90.2%) for patients treated with SSG. At 30 days, patients treated with L-AMB had different treatment success rates depending on the dose and number of doses given. Khalil et al. reported, a treatment success rate of 22–73% with single dose of 7.5 mg/kg (*n* = 20) and 33–100% with a single dose of 10 mg/kg (*n* = 22) [32]. Tamiru et al. [25] also reported that depending on the total amount of L-AMB taken, treatment success rates ranged from 80.2% in patients receiving a total dose of less than 24 mg/kg to 96.7% in those patients receiving 24–35 mg/kg. Treatment success rate was good in patients taking a combination of SSG and PM. In one study, 90.1% of VL patients taking SSG-PM were reported to be cured at EOT [27]. The treatment success rates of MA, miltefosine, and PM were 78.6–100% [30], 94.1% [29], and 66.7–96.7% [31], respectively.

At 6MFU, 80.7% of patients receiving SSG had treatment success. L-AMB showed different treatment success rates depending on the dose and number of doses [32]. Single doses of 7.5 mg/kg (*n* = 20) and 10 mg/kg (*n* = 22) gave treatment success rates of 11–64% and 33–100%, respectively. Another L-AMB treatment regimen was a 7-day course of 3 mg/kg (*n* = 37) which resulted in a treatment success rate of 71–100%. The treatment success rates of PM, MA, and miltefosine were reported by single studies and were found to be 75–96.6% [31], 80–100% [30], and 60% [29], respectively.

### VL mortality

**Fig. 4:**
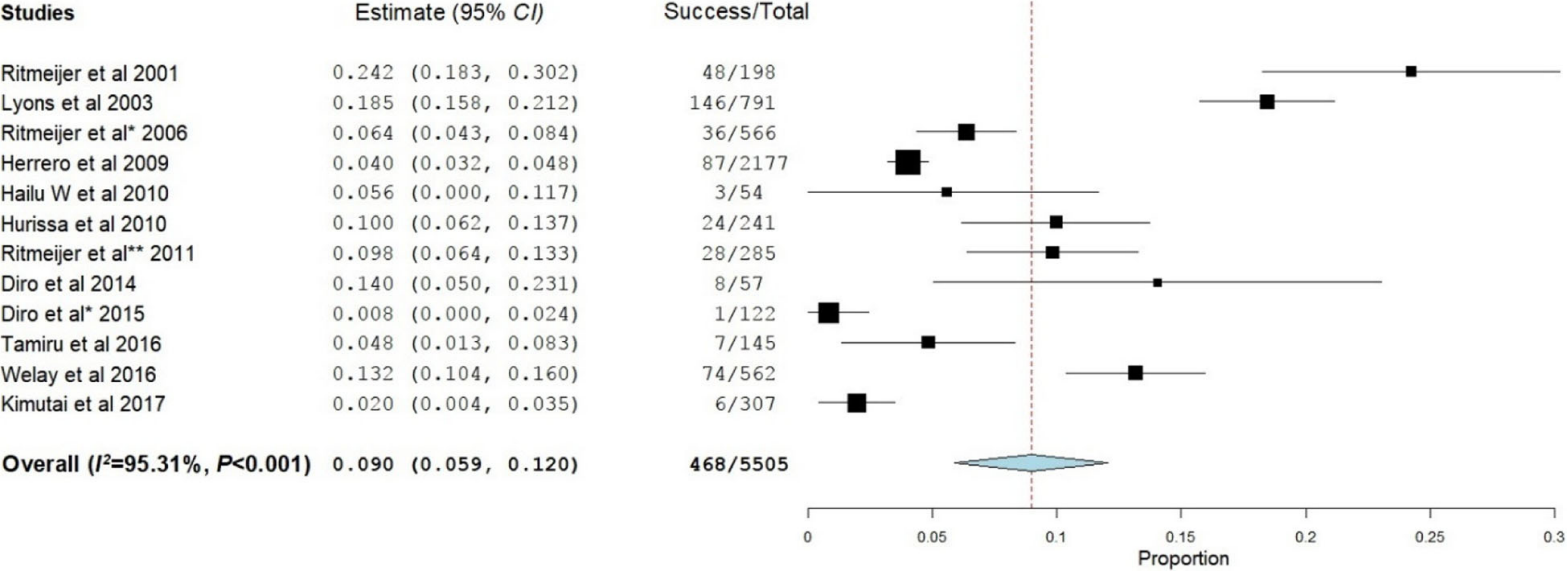
Mortality rate of visceral leishmaniasis patients at the end of the treatment

**Fig. 5:**
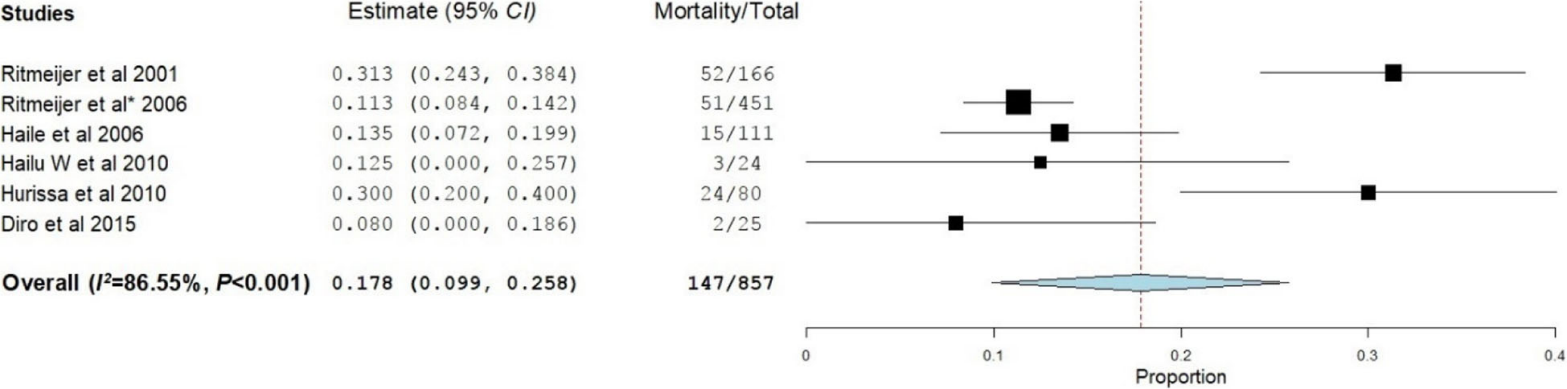
Mortality rate of visceral leishmaniasis patients at six months of follow-up

Twelve studies reported the mortality rate at the EOT [18, 20–30] while six studies reported a mortality rate at 6MFU [19, 21, 24, 28–30]. The overall mortality rates at EOT and 6MFU were 9.0% (95% *CI*: 5.9–12.0%, *P* < 0.001) (Fig. 4) and 17.8% (95% *CI*: 9.9–25.8%, *P* < 0.001), respectively (Fig. 5).

At EOT, the mortality rate among patients treated with SSG was 13.2% (95% *CI*: 5.7–20.8%, *P* < 0.001). Tamiru et al. [25] and Ritmeijer et al. [22] reported that, at EOT, the mortality rate among patients treated with L-AMB was 4.8%, ranging from 3.3 to 5.8% depending on the dose given. Mortality rates of miltefosine, MA, and SSG-PM were 2.8% [29], 6.25% [30], and 1.9% [27], respectively. At 6MFU, the mortality rate among patients treated with SSG rose to 19.7% (95% *CI*: 9.5–29.8%, *P* < 0.001) while mortality rate reached 5.9% for patients treated with miltefosine [29].

### VL treatment outcomes in HIV-infected and HIV-negative patients

Ten studies [18, 20–23, 25, 28–30, 32] assessed treatment outcomes in HIV-infected and/or HIV-negative VL patients at EOT while only five studies [21, 28–30, 32] assessed treatment outcomes at 6MFU. At EOT, the overall treatment success rate for HIV-infected patients was 66.8% (95% *CI*: 51.6–82.0%, *P* < 0.001) while it was 93.2% (95% *CI*: 89.8–96.5%, *P* < 0.001) for HIV-negative individuals. The overall treatment success rate at 6MFU for HIV-infected and HIV-negative patients was 42.5% (95% *CI*: 19.6–65.3%, *P* < 0.001) and 87.9% (95% *CI*: 80.7–95.2%, *P* < 0.001), respectively. The overall mortality rate at EOT for HIV-infected and HIV-negative patients was 14.4% (95% *CI*: 8.8–20.0%, *P* < 0.001) and 3.7% (95% *CI*: 2.2–5.2%, *P* = 0.082), respectively. At 6MFU, the overall mortality rate for HIV-infected patients was 29.9% (95% *CI*: 10.4–49.5%, *P* = 0.001) while it was 6.7% (95% *CI*: 0.8–12.5%, *P* = 0.015).

Treatment outcomes at EOT and 6MFU were compared between HIV-infected and HIV-negative VL patients. Except overall mortality rate at 6MFU, they failed to show a statistically significant difference (Tables 1 and 2). Patients with HIV infection showed more than 4-fold increase in overall mortality at 6MFU (Table 2).

**Table 1:** Visceral leishmaniasis-HIV co-infection and treatment success

	Studies	HIV- (Sample/ Total)	HIV+ (Sample/ Total)	*OR* (95% *CI*)	*P*-value	*I* ^*2*^
EOT						
Overall treatment success	6 studies [21, 22, 25, 28–30]	784/819	345/485	7.53 (4.93–11.50)	0.482	0.00%
SSG treatment success	3 studies [18, 28, 29]	392/411	93/119	6.09 (2.84–13.04)	0.272	23.09
6MFU						
Overall treatment success	4 studies [21, 28–30]	346/374	77/161	11.33 (6.78–18.91)	0.609	0.00%
SSG treatment success	2 studies [28, 29]	211/224	35/58	11.66 (4.13–31.53)	0.206	37.36

Note: *OR* odds ratio, *CI* confidence interval, *EOT* End of treatment, *6MFU* 6 months follow-up

**Table 2:** Visceral leishmaniasis -HIV co-infection and treatment failure and mortality

	Studies	HIV+ (Sample/ Total)	HIV- (Sample/ Total)	*OR* (95% *CI*)	*P*-value	*I* ^*2*^
EOT						
Overall treatment failure	5 studies [18, 21, 22, 28, 29]	67/473	0/781	20.35 (4.87–85.03)	0.600	0.00
SSG treatment failure	3 studies [18, 28, 29]	1/119	0/411	5.55 (0.68–45.57)	0.912	0.00
Overall mortality	6 studies [18, 20–22, 28, 29]	71/523	39/1029	4.14 (2.48–6.89)	0.216	29.16
SSG mortality	3 studies [18, 28, 29]	25/119	19/411	5.79 (2.39–14.01)	0.195	38.79
6MFU						
Overall treatment failure	3 studies [21, 28, 29]	43/158	7/362	12.65 (5.44–29.43)	0.641	0.00
SSG treatment failure	2 studies [28, 29]	7/58	1/224	18.39 (2.82–120.00)	0.489	0.00
Overall mortality	3 studies [21, 28, 29]	39/158	21/362	4.77 (1.30–17.43)	*	78.96
SSG mortality	2 studies [28, 29]	16/58	12/224	7.03 (1.32–37.33)	0.05	73.86

Note: *OR* odds ratio, *CI* confidence interval, *EOT* End of treatment, *6MFU* 6 months follow-up, *SSG* sodium stibogluconate, *p-value < 0.005

At EOT, HIV-infected patients treated with SSG had a 68.8% (95% *CI*: 47.1–90.5%, *P* < 0.001) treatment success and an 18.6% (95% *CI:* 7.5–29.6%, *P* = 0.006) mortality rates. Their HIV-negative counterparts had a 95.9% (95% *CI:* 93.7–98.0%, *P* = 0.290) and a 4.1% (95% *CI*: 2.0–6.3%, *P* = 0.290) treatment success and mortality rates, respectively. At 6MFU, HIV-infected patients treated with SSG had a 58.3% (95% *CI*: 31.0–85.7%, *P* < 0.030) treatment success and a 30.0% (95% *CI*: -2.8–62.8%, *P* = 0.005) mortality rates. Treatment success and mortality rates of their HIV-negative counterparts were 94.2% (95% *CI:* 91.2–97.3%, *P* = 0.775) and 5.4% (95% *CI*: 2.4–8.3%, *P* = 1.000), respectively. However, meta-analyses of studies that compared treatment outcomes between HIV-infected and HIV-negative patients who were treated with SSG did not show statistically significant differences (Tables 1 and 2).

Ritmeijer et al. [22] reported that treatment success (92.6% vs 59.5%) and mortality (6.4% vs 6.7%) rates at EOT among patients treated with 6 doses of L-AMB was better for HIV-negative patients than HIV-infected ones. Kimutai et al. [27] also reported better treatment success at EOT with SSG-PM among HIV-negative patients than HIV-infected ones. The overall treatment success rate was 90.1% but sub-group analysis of HIV-negative individuals gave a success rate of 93%. EOT treatment success rates for MA (58% vs 92.9–100%) [30] and miltefosine (88.9% vs 97.7%) [29], and mortality rates for miltefosine (4.8% vs 0.8%) [29] and L-AMB (6.7% vs 6.4%) [22] were also better for HIV-negative patients. Likewise, treatment success rates of MA (33.3% vs 100%) [30], and of miltefosine (46.0% vs 75.6%) and mortality rate of miltefosine (11.1% vs 0.8%) [29] were also better at 6MFU.

### Quality assessment and sensitivity analysis

The 10 observational studies [18–27] were assessed with the STROBE statement [16] while the five interventional studies [28–32] were assessed using the modified Jadad scale [17]. All of these studies were judged to be of high quality (Additional file 3).

**Fig. 6:**
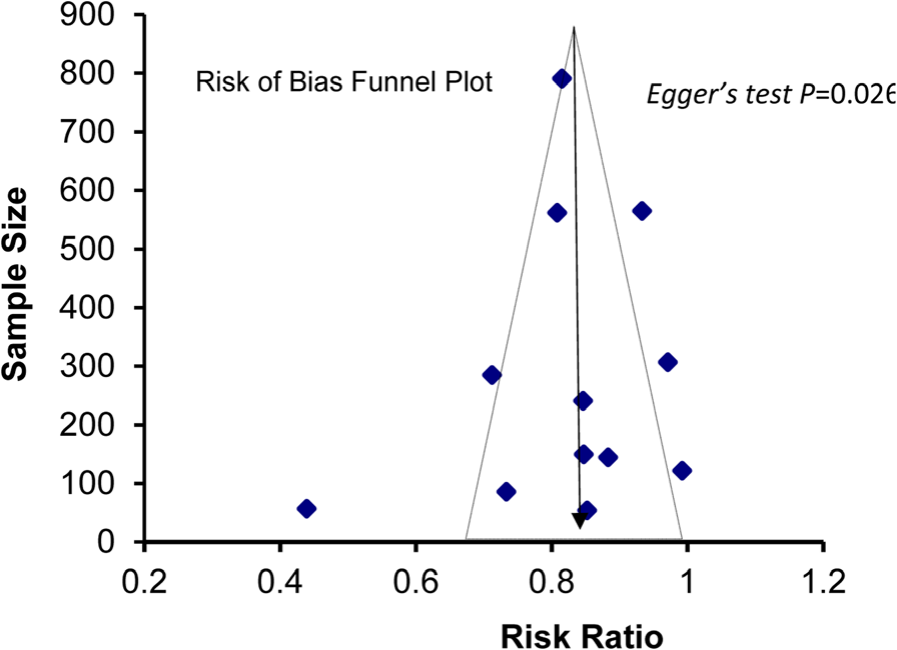
Funnel plot showing risk of bias

The meta-analysis was stratified based on the types of studies (observational and interventional) to investigate the overall treatment outcomes at EOT across the included studies. It revealed similar results between the observational studies (81.2%, 95% *CI*: 73.5–88.9%, *P* < 0.001, *I*^*2*^ = 97.53) [18–27] and interventional studies (84.2%, 95% *CI*: 77.1–91.4%*, P* < 0.001; *I*^*2*^ = 93.83) [28–32]. Publication bias was also assessed using a funnel plot and Egger’s test (Fig. 6).

## Discussion

Our systematic review identified 15 studies (from 2001 to 2017) that provided information for estimating treatment outcomes of VL in Ethiopia [18–32]. This systematic review and meta-analysis has shown wide ranges of treatment success rates ranging from 22.0% [32] to 99.2% [24]. The overall treatment success rate declined from 82.6% at EOT to 72.2% at 6MFU. The treatment success rates varied among patients mainly based on the type of antileishmanial medications used and based on the patients’ HIV status.

In the present study, the treatment success rate of pentavalent antimonials, SSG in particular, was found to be lower than the alternative treatments such as SSG-PM and multiple doses of L-AMB. Therefore, SSG should not be considered first-line treatment when either SSG-PM or multiple doses of L-AMB is available. Considering the advantages of shorter duration of treatment, reduced risk of drug resistance and adverse effects, and lower cost, WHO recommends the use of a combination of antileishmanial medications [33]. Considering this, the Ethiopian Standard Treatment Guideline recommends a 17 days course of the combination of SSG and PM to be used as a first-line treatment for VL. [11] A recent study by Kimutai et al. supports this recommendation where 90.1% of Ethiopian patients who used SSG-PM were reported to have treatment success at EOT [27]. This was higher than the 81.5% of treatment success rate of SSG. Like Ethiopia, other East African countries such as Uganda have also turned their attention to the combination of SSG and PM as a first line treatment for the management of VL. [34] On the other hand, the rates of treatment unresponsiveness to pentavalent antimonials in Asia were reported to reach as high as 60% [33].

In the present study, there were differences in treatment outcomes even among patients who received different dosages of L-AMB. Most importantly, patients who took single doses of L-AMB had unacceptably low rates of treatment success that can reach as low as 22%. This is different from the situation in India where a single dose of L-AMB as low as 3.75 mg/kg can give a cure rate close to 90% [35]. The optimal effective single-dose of L-AMB in Ethiopia is yet to be determined. Most patients who were treated with L-AMB have one or more of the following conditions: severe or critical illness, HIV-infection, and treatment relapse. Despite this, treatment success rates of L-AMB given in multiple doses (≥ 6 doses) were good. This makes multiple doses of L-AMB the preferred treatment option for patients with the conditions above.

Just below one-tenth of the study participants died during treatment. The number of patients who died almost doubled at 6MFU. These indicate the importance of patient follow-up after treatment completion even in those patients who were declared to be cured. Compared to patients receiving other antileishmanial agents, the mortality rate was higher in patients treated with SSG (13.2% at EOT and 19.7% at 6MFU). Previous studies reported the detrimental effects of HIV infection on mortality associated with VL. [36] Similarly, findings of the present study show that the overall mortality rate at 6MFU for patients with HIV infection increased 4.77 times than HIV-negative individuals. Associated with this, appropriate timing of antiretroviral therapy (ART) initiation needs to be investigated as earlier studies [37, 38] reported poor chances of survival with early ART-initiation among HIV-VL co-infected individuals. However, the present study did not assess this issue.

Few vaccines have been reported to be in clinical trials none of which have been shown to be cost-effective [39]. A vaccine that is effective, safe, and cost-effective against visceral leishmaniasis or leishmaniasis, in general, is yet to be available [40]. Until that day comes, we have to effectively use the medications at hand. A particular attention needs to be given to treatment optimization in the management of HIV-VL coinfection as treatment is complicated by several factors. Future research focusing to investigate the relationship between ART adherence and its implications on VL treatment outcomes is needed.

Our study has several strengths. In total, we identified 10 observational [18–27] and five interventional [28–32] studies which allowed us to pool results from 5852 patients with VL who were treated with a wide range of antileishmanial regimens. It also allowed us a comparison of outcomes among 1520 HIV-infected and negative patients treated for VL. Outcomes were confirmed clinically and/or parasitologically. Studies that were included in the final analyses were all found to be of high quality. We were also able to determine treatment outcomes both at EOT and 6MFU. Despite greater treatment failures during treatment in HIV patients, the findings from observational studies were consistent with those from trials, making the results more generalizable to practice under the conditions experienced in many VL endemic countries.

Nevertheless, our study was not without limitations. The study assessed treatment outcomes of VL in Ethiopia which makes is difficult to generalize the finding to other countries in East Africa. Some studies were conducted in multiple countries which made us unable to extract some important information. Not all studies reported treatment success, failure, and mortality at EOT and 6MFU which made us rely on studies that do.

## Conclusions

SSG alone has shown lower treatment efficacy in the management of VL when compared to SSG-PM and multiple doses of L-AMB. The combination of SSG with PM gave good treatment success rates with shorter duration of treatment. Hence, the combination of SSG with PM should be used preferentially over SSG monotherapy. This makes it rational to use it as a first-line medication in the country. On the other hand, single-doses of L-AMB showed low efficacy and should not be used in Ethiopia while multiple doses showed great efficacy especially among patients with complications, severe disease, HIV co-infection, and intolerance to the adverse effects of antimonials. Therefore, L-AMB should be used as a first-line treatment option in these patient populations. However, future studies should focus on determining the lowest effective dose of this drug. HIV-infected individuals had a worse prognosis than their HIV-negative counterparts. The association between ART and timing of ART on treatment outcomes should be investigated to determine the best time of ART administration in these patients. Lastly, treatment outcomes at 6MFU were found to inferior to at EOT and hence patients should be followed-up for possible relapses after declaring being cured.

## Additional files

Additional file 1: Excluded studies after review of full text articles. (DOCX 18 kb)

Additional file 2: Overview of the Visceral Leishmaniasis studies conducted in Ethiopia from 2001 to 2017 (*N* = 5851). (DOCX 17 kb)

Additional file 3: **Table S1.** Quality assessment of included studies. (DOCX 14 kb)

## Abbreviations

6MFU: Six months follow-up
ART: Antiretroviral therapy
*CI*: Confidence interval
EOT: End of treatment
HIV: Human immunodeficiency virus
L-AMB: Liposomal amphotericin b
MA: Meglumine antimoniate
*OR*: Odds ratio
PM: Paromomycin sulfate
PRISMA: Preferred Reporting Items for Systematic review and Meta-Analysis
SSG: Sodium stibogluconate
VL: Visceral leishmaniasis

## References

[1] Chappuis F, Sundar S, Hailu A, Ghalib H, Rijal S, Peeling RW (2007). Visceral leishmaniasis: what are the needs for diagnosis, treatment and control?. Nat Rev Microbiol.

[2] Desjeux P (2004). Leishmaniasis: current situation and new perspectives. Comp Immunol Microbiol Infect Dis.

[3] Hotez PJ, Alvarado M, Basáñez M-G, Bolliger I, Bourne R, Boussinesq M (2014). The global burden of disease study 2010: interpretation and implications for the neglected tropical diseases. PLoS Negl Trop Dis.

[4] Hotez PJ, Kamath A (2009). Neglected tropical diseases in sub-Saharan Africa: review of their prevalence, distribution, and disease burden. PLoS Negl Trop Dis.

[5] Hailu A, Gramiccia M, Kager P (2009). Visceral leishmaniasis in aba-Roba, South–Western Ethiopia: prevalence and incidence of active and subclinical infections. Ann Trop Med Parasitol.

[6] Leta S, Dao THT, Mesele F, Alemayehu G (2014). Visceral leishmaniasis in Ethiopia: an evolving disease. PLoS Negl Trop Dis.

[7] Alvar J, Yactayo S, Bern C (2006). Leishmaniasis and poverty. Trends Paraitol.

[8] van Griensven J, Diro E (2012). Visceral leishmaniasis. Infect Dis Clin N Am.

[9] Bern C, Maguire JH, Alvar J (2008). Complexities of assessing the disease burden attributable to leishmaniasis. PLoS Negl Trop Dis.

[10] World Health Organization (2010). Report of a meeting of the WHO Expert Committee on the Control of Leishmaniases.

[11] Food Medicine and Healthcare Administration and Control Authority of Ethiopia (2014). Ethiopia Standard Treatment Guidelines for General Hospital.

[12] Diro E, Lynen L, Ritmeijer K, Boelaert M, Hailu A, van Griensven J (2014). Visceral leishmaniasis and HIV coinfection in East Africa. PLoS Negl Trop Dis.

[13] Mahajan R, Das P, Isaakidis P, Sunyoto T, Sagili KD, Lima MA (2015). Combination treatment for visceral leishmaniasis patients coinfected with human immunodeficiency virus in India. Clin Infect Dis.

[14] Singh OP, Singh B, Chakravarty J, Sundar S (2016). Current challenges in treatment options for visceral leishmaniasis in India: a public health perspective. Infect Dis Poverty.

[15] Moher D, Liberati A, Tetzlaff J, Altman DG (2009). Preferred reporting items for systematic reviews and meta-analyses: the PRISMA statement. Ann Intern Med.

[16] Von Elm E, Altman DG, Egger M, Pocock SJ, Gøtzsche PC, Vandenbroucke JP (2007). The strengthening the reporting of observational studies in epidemiology (STROBE) statement: guidelines for reporting observational studies. Prev Med.

[17] Zhu Y, Wang C, Pang X, Li F, Chen W, Tan W (2014). Antibiotics are not beneficial in the management of category III prostatitis: a meta-analysis. Urol J.

[18] Lyons S, Veeken H, Long J (2003). Visceral leishmaniasis and HIV in Tigray, Ethiopia. Trop Med Int Health.

[19] Haile T, Anderson S (2006). Visceral leishmaniasis in northern Ethiopia. East Afr Med J.

[20] Herrero M, Orfanos G, Argaw D, Mulugeta A, Aparicio P, Parreño F, Bernal O, Rubens D, Pedraza J, Lima MA, Flevaud L (2009). Natural history of a visceral leishmaniasis outbreak in highland Ethiopia. Am J Trop Med Hyg.

[21] Hurissa Z, Gebre-Silassie S, Hailu W, Tefera T, Lalloo DG, Cuevas LE (2010). Clinical characteristics and treatment outcome of patients with visceral leishmaniasis and HIV co-infection in Northwest Ethiopia. Trop Med Int Health.

[22] Ritmeijer K, ter Horst R, Chane S, Aderie EM, Piening T, Collin SM (2011). Limited effectiveness of high-dose liposomal amphotericin B (AmBisome) for treatment of visceral leishmaniasis in an Ethiopian population with high HIV prevalence. Clin Infect Dis.

[23] Diro E, Lynen L, Mohammed R, Boelaert M, Hailu A, van Griensven J (2014). High parasitological failure rate of visceral leishmaniasis to sodium Stibogluconate among HIV co-infected adults in Ethiopia. PLoS Negl Trop Dis.

[24] Diro E, Lynen L, Gebregziabiher B, Assefa A, Lakew W, Belew Z (2015). Clinical aspects of paediatric visceral leishmaniasis in north-West Ethiopia. Trop Med Int Health.

[25] Tamiru A, Tigabu B, Yifru S, Diro E, Hailu A (2016). Safety and efficacy of liposomal amphotericin B for treatment of complicated visceral leishmaniasis in patients without HIV, north-West Ethiopia. BMC Infect Dis.

[26] Welay GM, Alene KA, Dachew BA (2017). Visceral leishmaniasis treatment outcome and its determinants in Northwest Ethiopia. Epidemiol Health..

[27] Kimutai R, Musa AM, Njoroge S, Omollo R, Alves F, Hailu A, Khalil EA, Diro E, Soipei P, Musa B, Salman K (2017). Safety and effectiveness of sodium stibogluconate and paromomycin combination for the treatment of visceral leishmaniasis in eastern Africa: results from a pharmacovigilance programme. Clin Drug Investig.

[28] Ritmeijer K, Veeken H, Melaku Y, Leal G, Amsalu R, Seaman J (2001). Ethiopian visceral leishmaniasis: generic and proprietary sodium stibogluconate are equivalent; HIV co-infected patients have a poor outcome. Trans R Soc Trop Med Hyg.

[29] Ritmeijer K, Dejenie A, Assefa Y, Hundie TB, Mesure J, Boots G (2006). A comparison of miltefosine and sodium stibogluconate for treatment of visceral leishmaniasis in an Ethiopian population with high prevalence of HIV infection. Clin Infect Dis.

[30] Hailu W, Weldegebreal T, Hurissa Z, Tafes H, Omollo R, Yifru S (2010). Safety and effectiveness of meglumine antimoniate in the treatment of Ethiopian visceral leishmaniasis patients with and without HIV co-infection. Trans R Soc Trop Med Hyg.

[31] Hailu A, Musa A, Wasunna M, Balasegaram M, Yifru S, Mengistu G (2010). Geographical variation in the response of visceral leishmaniasis to paromomycin in East Africa: a multicentre, open-label, randomized trial. PLoS Negl Trop Dis.

[32] Khalil EA, Weldegebreal T, Younis BM, Omollo R, Musa AM, Hailu W (2014). Safety and efficacy of single dose versus multiple doses of AmBisome® for treatment of visceral leishmaniasis in eastern Africa: a randomised trial. PLoS Negl Trop Dis.

[33] World Health Organization (WHO) (2010). Control of the leishmaniasis: report of a meeting of the WHO expert committee on the control of Leishmaniases, Geneva, 22–26 March 2010.

[34] Olobo-Okao J, Sagaki P (2014). Leishmaniasis in Uganda: historical account and a review of the literature. Pan Afr Med J.

[35] Rodrigo C, Weeratunga P, Fernando SD, Rajapakse S (2017). Amphotericin B for treatment of visceral leishmaniasis; a systematic review and meta-analysis of prospective, comparative clinical studies including dose ranging studies. Clin Microbiol Infect.

[36] Monge-Maillo B, Lopez-Velez R (2016). Treatment options for visceral leishmaniasis and HIV coinfection. AIDS Rev.

[37] Aderie EM, Diro E, Zachariah R, da Fonseca MS, Abongomera C, Dolamo BL, Ritmeijer K (2017). Does timing of antiretroviral treatment influence treatment outcomes of visceral leishmaniasis in Northwest Ethiopia?. Trans R Soc Trop Med Hyg.

[38] Burza S, Mahajan R, Sinha PK, van Griensven J, Pandey K, Lima MA, Sanz MG, Sunyoto T, Kumar S, Mitra G, Kumar R (2014). Visceral leishmaniasis and HIV co-infection in Bihar, India: long-term effectiveness and treatment outcomes with liposomal amphotericin B (AmBisome). PLoS Negl Trop Dis.

[39] Gillespie PM, Beaumier CM, Strych U, Hayward T, Hotez PJ, Bottazzi ME (2016). Status of vaccine research and development of vaccines for leishmaniasis. Vaccine.

[40] Sundar S, Singh A (2016). Recent developments and future prospects in the treatment of visceral leishmaniasis. Ther Adv Infect Dis.

