# Supplementary material for: Treatment outcomes of visceral leishmaniasis in Ethiopia from 2001 to 2017: a systematic review and meta-analysis
Source: Infect Dis Poverty. 2018 Oct 19;7:108. doi: 10.1186/s40249-018-0491-7 (PMC6194743; doi:10.1186/s40249-018-0491-7)
Supplement: Supplementary file 1 — Excluded studies after review of full text articles. (DOCX 18 kb) [file 40249_2018_491_MOESM1_ESM.docx]

# **Additional file 1: Excluded studies after review of full text articles**

|  | **Name** | **Title** | **Journal** | **Reason** |
| --- | --- | --- | --- | --- |
| 1 | Abongomera C et al 2017 | The Risk and Predictors of Visceral Leishmaniasis Relapse in Human Immunodeficiency Virus Co-infected Patients in Ethiopia: A Retrospective Cohort Study | Clin. Infect. Dis. | Measured only incidence of relapse at different times |
| 2 | Abongomera C et al 2017 | Development and external validation of a clinical prognostic score for death in visceral leishmaniasis patients in a high HIV coinfection burden area in Ethiopia | PLoS One | Treatment outcomes not assessed |
| 3 | Aderie E et al 2017 | Does timing of antiretroviral treatment influence treatment outcomes of visceral leishmaniasis in Northwest Ethiopia? | Trans R Soc Trop Med Hyg | Treatment outcomes not consistently assessed at EOT |
| 4 | Alemayehu M et al 2017 | Prevalence of Human Immunodeficiency Virus and associated factors among Visceral Leishmaniasis infected patients in Northwest Ethiopia: a facility based cross-sectional Study | BMC Infect Dis | Treatment outcomes not assessed |
| 5 | Alvar J et al 2008 | The Relationship between Leishmaniasis and AIDS: the Second 10 Years | Clin Microbiol Rev | Treatment outcomes not assessed |
| 6 | Berhe N et al 1994 | Relapse in Ethiopian visceral leishmaniasis (VL) patients after therapy with pentavalent antimonials: a ten year observation | Acta Trop | Studied relapse cases over the years |
| 7 | Berhe N et al 1995 | Ethiopian visceral leishmaniasis patients co-infected with human immunodeficiency virus | T Roy Soc Trop Med H | Less than 10 patients included; patients received varying doses of SSG |
| 8 | Berhe N et al 2001 | Electrocardiographic findings in Ethiopians on pentavalent antimony therapy for visceral leishmaniasis | East Afr Med J | Scope is not up to the aim of the study |
| 9 | Berhe N et al 2001 | Inter-current and nosocomial infections among visceral leishmaniasis patients in Ethiopia: an observational study | Acta Trop | Studied inter-current and nosocomial infections |
| 10 | Berman J et al 2008 | Treatment of leishmaniasis with miltefosine: 2008 status | J Drug Metab Toxicol | Not an original article |
| 11 | Beshah AM et al 2011 | Clinical Manifestations and Anthropometric Profiles of Visceral Leishmaniasis in Selected Centers in Ethiopia | Unpublished Literature | Didn’t specify the antileishmanial drug used |
| 12 | Chappuis F 2011 | High Mortality among Older Patients Treated with Pentavalent Antimonials for Visceral Leishmaniasis in East Africa and Rationale for Switch to Liposomal Amphotericin B | Antimicrob. Agents Chemother. | Not an original article |
| 13 | Diro E et al 2014 | Visceral Leishmaniasis and HIV Coinfection in East Africa | PLoS Negl. Trop. Dis. | Not an original article |
| 14 | Diro E et al. 2014 | Impact of the Use of a Rapid Diagnostic Test for Visceral Leishmaniasis on Clinical Practice in Ethiopia: A Retrospective Study | PLoS Negl. Trop. Dis. | Treatment outcomes not assessed |
| 15 | Diro E et al 2015 | Atypical manifestations of visceral leishmaniasis in patients with HIV in north Ethiopia: a gap in guidelines for the management of opportunistic infections in resource poor settings | Lancet Infect Dis | Not original article |
| 16 | Diro E et al 2017 | Long-term clinical outcomes in visceral leishmaniasis-HIV co-infected patients during and after pentamidine secondary prophylaxis in Ethiopia: a single-arm clinical trial | Clin. Infect. Dis. | Treatment outcomes not assessed |
| 17 | Edwards T et al 2011 | Single-dose liposomal amphotericin B (AmBisome®) for the treatment of Visceral  Leishmaniasis in East Africa: study protocol for a randomized controlled trial | Trials | Study protocol |
| 18 | Huruy K et al 2008 | Leishmaniasis in HIV- infected individuals | Pharmacologyonline | Treatment outcomes not assessed |
| 19 | Kebede T et al 2005 | Indirect hemagglutination assay for diagnostic and epidemiological studies of Visceral leishmaniasis in Ethiopia | Unpublished Literature | Treatment outcomes not assessed |
| 20 | Mengesha B et al 2014 | Prevalence of malnutrition and associated risk factors among adult visceral leishmaniasis  patients in Northwest Ethiopia: a cross sectional study | BMC res notes | Treatment outcomes not assessed |
| 21 | Mengistu et al. 2016 | Ethiopia and its steps to mobilize resources to achieve 2020 elimination and control goals for neglected tropical diseases: Spider webs joined can tie a lion | Int. Health | Not an original article |
| 22 | Musa A et al 2012 | Sodium Stibogluconate (SSG) & Paromomycin Combination Compared to SSG for Visceral Leishmaniasis in East Africa: A Randomized Controlled Trial | PLoS Negl. Trop. Dis. | Unable to extract Ethiopian data |
| 23 | ter Horst R et al 2008 | Concordant HIV Infection and Visceral Leishmaniasis in Ethiopia: The Influence of Antiretroviral Treatment and Other Factors on Outcome | Clin. Infect. Dis. | Used variable follow-up periods |
| 24 | van Griensven J et al 2012 | Visceral Leishmaniasis | [Infect Dis Clin North Am](https://www.ncbi.nlm.nih.gov/nlmcatalog?term=%22Infect+Dis+Clin+North+Am%22%5Bta%5D) | Treatment outcomes not assessed |
| 25 | van Griensven J et al. 2014 | A Screen-and-Treat Strategy Targeting Visceral Leishmaniasis in HIV-Infected Individuals in Endemic East African Countries: The Way Forward? | PLoS Negl. Trop. Dis. | Not an original article |
| 26 | van Griensven J et al. 2014 | Combination therapy for visceral leishmaniasis | Lancet infect Dis | Not an original article |
