# Supplementary material for: Treatment outcomes of visceral leishmaniasis in Ethiopia from 2001 to 2017: a systematic review and meta-analysis
Source: Infect Dis Poverty. 2018 Oct 19;7:108. doi: 10.1186/s40249-018-0491-7 (PMC6194743; doi:10.1186/s40249-018-0491-7)
Supplement: Supplementary file 2 — Overview of the Visceral Leishmaniasis studies conducted in Ethiopia from 2001 to 2017 (N = 5851). (DOCX 17 kb) [file 40249_2018_491_MOESM2_ESM.docx]

**Additional file 2. Overview of the Visceral Leishmaniasis studies conducted in Ethiopia from 2001-2017 (N=**5851**)**

| **Study (citation)** | **Journal** | **Study design** | **Location** | **Study duration** | **Sample size (EOT)** | **Sample size (6MFU)** | **Mean/ median age (years)** | **Treatment options** | **Daily doses** | **Total number of doses** |
| --- | --- | --- | --- | --- | --- | --- | --- | --- | --- | --- |
| Ritmeijer K et al 2001(28) | Trans R Soc Trop Med Hyg | Randomized Controlled Trial | Densha | 6 months | 198 | 166 | 28* | SSG | 20 mg/kg | 30 |
| Lyons S et al 2003 (18) | TM & IH | Retrospective Cohort Study |  | 18 months | 791 | - | 25^#^ | SSG | 20 mg/kg | 30 |
| Haile T et al. 2006 (19) | East Afr Med J | Retrospective Cohort Study | Axum | 20 months | - | 111 | 27^#^ | SSG | 20 mg/kg | 30 |
| Ritmeijer K et al 2006 (29) | Clin. Infect. Dis. | Randomized Controlled Trial | Humera |  | 566 | 451 | 29.3* | Miltefosine; SSG | 100 mg; 20 mg/kg | 28; 30 |
| Herrero M et al 2009 (20) | Am. J. Trop. Med. Hyg | Retrospective Cohort Study | Libo Kemkem | 19 months | 2177 | - | - | SSG; L-AMB; SSG plus L-AMB | - | - |
| Hailu W et al 2010 (30) | Trans R Soc Trop Med Hyg | Single-arm Interventional Study | Gondar, Arbaminch | 13 months | 54 | 24 | 21.07* | MA | 20 mg/kg | 30 |
| Hailu A et al 2010 (31) | PLoS Negl. Trop. Dis. | Randomized Controlled Trial | Gondar, Arbaminch | 42 months | 150 | 138 | - | SSG; PM | 20 mg/kg; 15 mg/kg | 30; 21 |
| Hurissa Z et al 2010 (21) | TM & IH | Retrospective Cohort Study | Gondar, Humera | 24 months | 241 | 80 | 26.8* | SSG; MA; L-AMB | 20 mg/kg; 20 mg/kg; 3 mg/kg | 28-30; 28-30; 6-10 |
| Ritmeijer K et al 2011 (22) | Clin. Infect. Dis. | Retrospective Cohort Study | Humera, Abdurafi | 24 months | 285 | - | - | L-AMB | 5 mg/kg | 6 |
| Diro E et al 2014 (23) | PLoS Negl. Trop. Dis. | Retrospective Cohort Study | Gondar | 26 months | 57 | - | 32^#^ | SSG | 20 mg/kg | 30 |
| Khalil E et al 2014 (32) | PLoS Negl. Trop. Dis. | Randomized Controlled Trial | Gondar, Arbaminch | 17 months | 86 | 79 | - | L-AMB | 3 mg/kg; 7.5 mg/kg; 10 mg/kg; 12.5 mg/kg; 15 mg/kg | 7; 1; 1; 1; 1 |
| Diro E et al 2015(24) | TM & IH | Prospective Cohort Study | Gondar, Humera | 12 months | 122 | 25 | 8.5^#^ | SSG; SSG-PM; L-AMB | 20 mg/kg; 20 mg/kg plus 15 mg/kg; 3-5 mg/kg | 30; 17; 6-8 |
| Tamiru A et al 2016 (25) | BMC Infect Dis | Retrospective Cohort Study | Gondar | 72 months | 145 | - | 25^#^ | L-AMB | 3-5 mg/kg | 6 |
| Welay GM et al 2016 (26) | Epidemiol Health | Retrospective Cohort Study | Humera | 30 months | 562 | - | - | SSG | 20 mg/kg | 30 |
| Kimutai R et al 2017 (27) | Clin Drug Investig | Prospective Cohort Study | Gondar, Arbaminch, Abdurafi | 18 months | 307 | - | 22* | SSG-PM | 20 mg/kg plus 15 mg/kg | 17 |

*mean age; ^#^median age
