# Supplementary material for: Treatment outcomes of visceral leishmaniasis in Ethiopia from 2001 to 2017: a systematic review and meta-analysis
Source: Infect Dis Poverty. 2018 Oct 19;7:108. doi: 10.1186/s40249-018-0491-7 (PMC6194743; doi:10.1186/s40249-018-0491-7)
Supplement: Supplementary file 3 — Table S1. Quality assessment of included studies. (DOCX 14 kb) [file 40249_2018_491_MOESM3_ESM.docx]

**Table S1. Quality assessment of included studies**

| **Study** | **Type** | **Tool** | **Score** | **Percentage** |
| --- | --- | --- | --- | --- |
| Ritmeijer K et al 2001 | Randomized Controlled Trial | Modified Jadad | 5/8 | - |
| Lyons S et al 2003 | Retrospective Cohort Study | STROBE | 19/22 | 86.36% |
| Haile T et al 2006 | Retrospective Cohort Study | STROBE | 19/22 | 86.36% |
| Ritmeijer K et al 2006 | Randomized Controlled Trial | Modified Jadad | 6/8 | - |
| Herrero M et al 2006 | Retrospective Cohort Study | STROBE | 19/22 | 86.36% |
| Hailu W et al 2010 | Single-arm Interventional Study | STROBE | 18/22 | 81.82% |
| Hailu A et al 2010 | Randomized Controlled Trial | Modified Jadad | 6/8 | - |
| Hurissa Z et al 2010 | Retrospective Cohort Study | STROBE | 19/22 | 86.36% |
| Ritmeijer et al 2011 | Retrospective Cohort Study | STROBE | 20/22 | 90.91% |
| Diro E et al 2014 | Retrospective Cohort Study | STROBE | 18/22 | 81.82% |
| Khalil E et al 2014 | Randomized Controlled Trial | Modified Jadad | 6/8 | - |
| Diro E et al 2015 | Prospective Cohort Study | STROBE | 17/22 | 77.27% |
| Tamiru A et al 2016 | Retrospective Cohort Study | STROBE | 19/22 | 86.36% |
| Welay GM et al 2016 | Retrospective Cohort Study | STROBE | 18/22 | 81.82% |
| Kimutai R et al 2017 | Prospective Cohort Study | STROBE | 19/22 | 86.36% |
